# Implementation of a risk-stratified perioperative safety pathway for children undergoing elective ERCP: a single-center real-world retrospective cohort study

**DOI:** 10.3389/fped.2026.1900187

**Published:** 2026-08-14

**Authors:** Tian-Hang Wu, Yun-Xia Zhang, Shi-Qin Qi, Ran Tang

**Affiliations:** Department of General Surgery, Anhui Provincial Children’s Hospital, Hefei, Anhui, China

**Keywords:** adverse events, early warning, nasobiliary drainage, pediatric ERCP, perioperative safety, real-world study, risk stratification

## Abstract

**Background:**

Endoscopic retrograde cholangiopancreatography (ERCP) is increasingly used in children with pancreaticobiliary diseases. Although pediatric ERCP is feasible in experienced centers, perioperative adverse events, delayed recognition of early warning signs, and drainage-related problems remain important safety concerns. This study evaluated the real-world implementation of a risk-stratified perioperative safety pathway in children undergoing elective ERCP.

**Methods:**

This single-center retrospective historical cohort study included consecutive children who underwent elective ERCP at Anhui Provincial Children's Hospital between June 2022 and December 2025. Children treated from June 2022 to June 2023 received routine perioperative management, whereas those treated from July 2023 to December 2025 were managed under a risk-stratified perioperative safety pathway. The pathway incorporated structured preoperative risk assessment, procedure-related handover, early warning-based postoperative monitoring, standardized nasobiliary drainage management, and family-centered perioperative education. The primary outcome was the proportion of children who experienced at least one prespecified in-hospital perioperative adverse event. Secondary outcomes included postoperative recovery indicators and caregiver-reported care experience.

**Results:**

A total of 63 children were included, with 30 in the routine management group and 33 in the risk-stratified pathway group. Baseline demographic and disease characteristics were comparable between groups. Compared with routine management, the pathway group had a shorter time to first flatus (20.3 ± 5.1 vs. 27.5 ± 6.2 h, *P* < 0.01), earlier resumption of oral intake (33.6 ± 7.9 vs. 44.2 ± 8.7 h, *P* < 0.01), and a shorter length of hospital stay (5.2 ± 1.8 vs. 7.5 ± 2.1 days, *P* < 0.01). The proportion of children with at least one in-hospital perioperative adverse event was lower in the pathway group than in the routine management group (24.2% vs. 53.3%, *P* = 0.017). Nasobiliary drainage-related complications were less frequently observed in the pathway group (6.1% vs. 23.3%, *P* = 0.046).

**Conclusions:**

In this single-center retrospective historical cohort, implementation of a risk-stratified perioperative safety pathway was associated with faster postoperative recovery, fewer in-hospital perioperative adverse events, and better caregiver-reported care experience. Exploratory findings for individual adverse-event components, including nasobiliary drainage-related complications, should be interpreted cautiously because of the small sample size, multiple comparisons, and potential temporal confounding. Prospective multicenter studies are needed to validate these associations.

## 1 Introduction

Endoscopic retrograde cholangiopancreatography (ERCP) is an established endoscopic procedure for the diagnosis and treatment of pancreaticobiliary diseases. Although ERCP has long been used in adult practice, its use in children has increased with advances in pediatric anesthesia, endoscopic accessories, and specialized endoscopy teams (1). In pediatric patients, ERCP is now performed for a range of indications, including choledocholithiasis, biliary strictures, pancreatic duct disorders, and congenital pancreaticobiliary anomalies (2–4).

Despite its clinical value, ERCP remains an invasive and technically demanding procedure. Procedure-related adverse events may include post-ERCP pancreatitis, hyperamylasemia, infection, bleeding, perforation, respiratory events, anesthesia-related complications, and drainage-related problems (5, 6). Pediatric studies and systematic reviews have reported favorable technical success rates but clinically relevant complication rates, with post-ERCP pancreatitis being among the most common adverse events (2, 3). Improving perioperative safety therefore remains an important issue in pediatric ERCP.

Children undergoing ERCP have specific perioperative vulnerabilities. Younger children may have difficulty describing abdominal pain, nausea, or discomfort, and postoperative deterioration may initially be reflected only by changes in behavior, vital signs, feeding tolerance, or drainage characteristics. Children may also have limited ability to cooperate with tube protection, body positioning, and symptom reporting. These features increase the importance of structured observation and timely response during the early postoperative period.

Most previous studies on pediatric ERCP have focused on indications, technical success, procedural risk factors, and endoscopist-related outcomes (7). Less attention has been paid to the perioperative safety system surrounding the procedure. In clinical practice, important warning signs, such as persistent abdominal pain, vomiting, fever, hypoxia, abnormal drainage, tube twisting, displacement, or obstruction, are often first identified after the procedure. A structured pathway linking risk stratification, procedural handover, early-warning monitoring, device management, escalation criteria, and caregiver participation may therefore be useful in pediatric ERCP.

Risk assessment and early identification are central components of current ERCP safety strategies. Major endoscopy society guidelines emphasize the need to identify patients at risk for post-ERCP pancreatitis and to apply appropriate prevention and monitoring strategies (8, 9). In parallel, pediatric early warning systems have been developed to support earlier recognition of clinical deterioration in hospitalized children (10, 11). These concepts provide a rationale for a procedure-specific perioperative safety pathway for pediatric ERCP.

In our department, a risk-stratified perioperative safety pathway was introduced into routine practice for children undergoing elective ERCP. The pathway was designed to move perioperative management from experience-based observation toward structured, risk-oriented care. It included preoperative risk assessment, procedure-related risk handover, early postoperative warning-sign monitoring, standardized nasobiliary drainage care, caregiver education, and predefined escalation criteria. The present study retrospectively evaluated whether implementation of this pathway was associated with improved postoperative recovery, fewer in-hospital adverse events, and improved caregiver-reported care experience.

## 2 Materials and methods

### 2.1 Study design and setting

This was a single-center retrospective historical cohort study conducted in the Department of General Surgery at Anhui Provincial Children's Hospital. The study was designed and reported with reference to the Strengthening the Reporting of Observational Studies in Epidemiology (STROBE) statement (12). The study was approved by the Ethics Committee of Anhui Provincial Children's Hospital (Approval No. EYLL-2025-077). The requirement for informed consent was waived because of the retrospective nature of the study and the use of routinely collected clinical data.

### 2.2 Study population

Children who underwent elective ERCP between June 2022 and December 2025 were consecutively screened for eligibility. Elective ERCP was defined as a planned, non-emergency procedure performed after routine preoperative evaluation, anesthesia assessment, and perioperative preparation.

Patients were included if they met all of the following criteria: (1) underwent elective ERCP during the study period; (2) were younger than 18 years of age; (3) had complete perioperative medical and safety-management records; and (4) completed in-hospital observation from ERCP to discharge. Patients were excluded if they: (1) underwent emergency ERCP; (2) had incomplete clinical records or missing key outcome data; (3) were transferred to another institution after ERCP; or (4) were discharged before completion of in-hospital perioperative observation.

### 2.3 Group allocation

Patients were categorized into two groups according to the perioperative management model routinely used during different historical periods. Children treated between June 2022 and June 2023 received routine perioperative management and were assigned to the routine management group. Children treated between July 2023 and December 2025 were managed under the risk-stratified perioperative safety pathway and were assigned to the pathway group.

Group allocation was based on real-world implementation of departmental practice rather than random assignment. No prospective intervention or randomization was performed. All ERCP procedures were performed by the same clinical team according to institutional procedural standards. No major institutional changes in ERCP indications, anesthesia protocols, or postoperative medical treatment were identified during the study period. Nevertheless, because the two groups were defined by historical periods, potential temporal bias related to increasing team experience and unmeasured practice changes could not be completely excluded.

### 2.4 Technical performance of ERCP

All ERCP procedures were performed in a dedicated endoscopy setting by an experienced pediatric pancreaticobiliary endoscopy team, with support from anesthesiologists and perioperative nursing staff. Before the procedure, clinical indications, laboratory results, imaging findings, anesthesia risk, and the expected need for drainage were reviewed by the treating team. Caregivers received routine explanations regarding fasting, anesthesia, procedural objectives, potential complications, and postoperative observation requirements.

Procedures were performed under general anesthesia according to institutional pediatric anesthesia practice. After induction and airway management, the child was positioned appropriately for ERCP, usually in the prone or left lateral position depending on age, body size, anesthesia requirements, and procedural feasibility. Continuous monitoring included oxygen saturation, heart rate, blood pressure, respiratory status, and other anesthesia-related parameters. The duodenoscope was advanced to the descending duodenum, and the major papilla was identified. Selective cannulation of the common bile duct or pancreatic duct was attempted using standard guidewire-assisted techniques when appropriate. Contrast injection and fluoroscopic imaging were used to delineate ductal anatomy, identify stones, strictures, ductal dilatation, or pancreaticobiliary anomalies, and guide therapeutic decisions.

Therapeutic maneuvers were selected according to the underlying disease and intraoperative findings. Endoscopic sphincterotomy or papillary balloon dilation was performed when indicated. Stone extraction was performed using baskets or balloon catheters, and repeated duct clearance was guided by cholangiographic findings. Biliary stent placement or nasobiliary drainage was selected when ongoing drainage, decompression, or short-term postoperative observation of bile flow was considered necessary. During the procedure, unnecessary pancreatic duct manipulation and repeated contrast injection were avoided whenever possible to reduce procedure-related risk. Procedure duration, cannulation difficulty, pancreatic duct manipulation, sphincterotomy, stone extraction, stent placement, and nasobiliary drainage placement were communicated to the ward team after the procedure as risk-relevant handover information.

After ERCP, children recovered under anesthesia and endoscopy team supervision before returning to the ward. Postoperative management included observation for abdominal pain, vomiting, fever, abdominal distension, respiratory events, altered mental status, bleeding, and signs suggesting pancreatitis, infection, perforation, or drainage-related problems. The technical details of ERCP and post-procedure risk information were incorporated into the perioperative safety pathway to guide monitoring intensity and escalation decisions.

### 2.5 Routine perioperative management

Children in the routine management group received standard perioperative management according to institutional practice for pediatric ERCP. This included routine preoperative assessment, fasting preparation, anesthesia evaluation, caregiver instruction, postoperative monitoring of vital signs and symptoms, drainage tube observation when applicable, dietary guidance, and discharge education. Postoperative abnormalities were reported to the responsible physician according to routine clinical judgment.

### 2.6 Risk-stratified perioperative safety pathway

**Figure 1:**
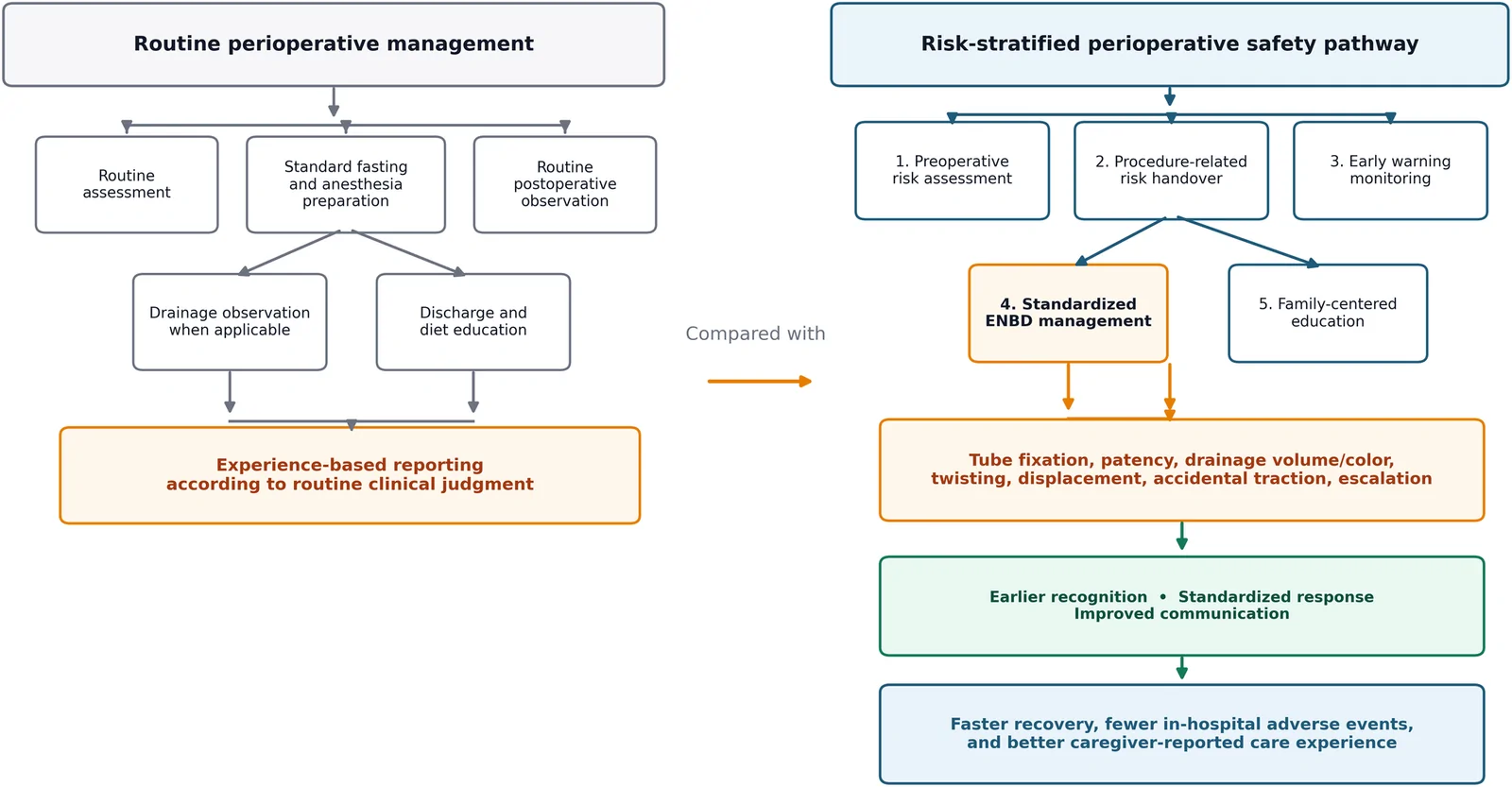
Perioperative management models for pediatric ERCP. Schematic comparison of routine perioperative management and the risk-stratified perioperative safety pathway for pediatric ERCP. The pathway integrated preoperative risk assessment, procedure-related handover, early warning monitoring, standardized nasobiliary drainage management, and family-centered education to support earlier recognition, standardized response, and improved perioperative communication.

The risk-stratified perioperative safety pathway was implemented as a structured safety-management process for pediatric ERCP. The pathway was not designed as an isolated nursing intervention, but as a multidisciplinary perioperative safety strategy involving surgeons/endoscopists, anesthesiologists, ward physicians, nurses, patients, and caregivers. Figure 1 summarizes the standard perioperative management model and the five core components of the risk-stratified pathway, including standardized nasobiliary drainage management.

#### 2.6.1 Preoperative risk assessment

Before ERCP, patients were evaluated for potential perioperative risk factors, including prior pancreatitis, suspected biliary infection, coagulation abnormalities, poor nutritional status, young age, poor cooperation, anticipated difficult procedure, and expected need for postoperative drainage. Children with potential risk factors received individualized perioperative plans and focused caregiver education.

#### 2.6.2 Procedure-related risk communication

After ERCP, procedure-related information was communicated to the ward team as part of a structured handover. Risk-relevant procedural factors included prolonged procedure duration, difficult cannulation, repeated pancreatic duct manipulation, pancreatic duct opacification, endoscopic sphincterotomy, stone extraction, stent placement, and nasobiliary drainage placement. This handover allowed postoperative monitoring intensity to be tailored according to procedural risk.

#### 2.6.3 Early postoperative warning-sign monitoring

During the first 6 h after ERCP, children were monitored using predefined warning indicators. These included abdominal pain, vomiting, abdominal distension, fever, changes in heart rate or blood pressure, oxygen desaturation, altered mental status, abnormal drainage volume or color, and suspected tube displacement or obstruction. Abnormal findings were escalated to the responsible physician according to predefined criteria.

#### 2.6.4 Standardized nasobiliary drainage management

For children with nasobiliary drainage, standardized device management was emphasized. Tube fixation, tube patency, drainage volume, drainage color, tube twisting, displacement, compression, and accidental traction were checked regularly. Caregivers were instructed to avoid pulling or compressing the tube, maintain an appropriate body position, observe drainage characteristics, and report abnormalities immediately. Tube-related problems were escalated and managed promptly to reduce obstruction, displacement, or twisting.

The decision to use nasobiliary drainage, biliary stenting, or no postoperative drainage was made by the endoscopist according to the underlying disease and intraoperative findings. Nasobiliary drainage was generally considered when temporary external drainage, direct observation of bile characteristics, short-term irrigation, or postoperative assessment of drainage patency was required. Biliary stenting was generally considered when sustained internal drainage was required without the need for external bile monitoring. Factors considered included biliary infection, residual stones or sludge, biliary obstruction or stricture, ductal anatomy, and adequacy of biliary drainage.

#### 2.6.5 Family-centered perioperative education

Caregivers were actively involved in perioperative safety management. Education focused on fasting and diet resumption, pain and fever reporting, tube protection, recognition of warning symptoms, and cooperation with postoperative observation. This component was consistent with family-centered care principles, which emphasize partnership, communication, respect, and caregiver participation in pediatric care (13, 14).

### 2.7 Outcomes

The primary outcome was the proportion of unique patients who experienced at least one prespecified in-hospital perioperative adverse event. The composite outcome included post-ERCP pancreatitis, hyperamylasemia, biliary infection, bleeding, perforation, respiratory complications, anesthesia-related adverse reactions, and nasobiliary drainage-related complications. Individual adverse-event categories were analyzed as exploratory components of the composite outcome.

Secondary outcomes included time to first flatus, time to resumption of oral intake, length of hospital stay, and caregiver-reported care experience before discharge. Post-ERCP pancreatitis was defined according to clinical symptoms and biochemical evidence of pancreatic injury after ERCP, as recorded in the medical record. Hyperamylasemia was defined as an elevated serum amylase level after ERCP without sufficient clinical evidence to meet the diagnostic criteria for post-ERCP pancreatitis. Nasobiliary drainage-related complications included tube obstruction, displacement, twisting, or accidental traction requiring clinical attention.

### 2.8 Caregiver-reported care experience

Caregiver-reported care experience was assessed before discharge using a structured questionnaire adapted from the Newcastle Satisfaction with Nursing Scales, which has been used to evaluate patient experience and satisfaction with care (15). The questionnaire assessed several domains, including professional care, communication and health education, psychological support, attitude and responsiveness, and overall care experience. Scores were recorded using a 5-point Likert scale, with higher scores indicating better caregiver-reported experience.

### 2.9 Statistical analysis

Statistical analyses were performed using SPSS version 27.0. Continuous variables were tested for normality using the Shapiro–Wilk test. Normally distributed continuous variables are presented as mean ± standard deviation and were compared using independent-samples t tests. Non-normally distributed variables are presented as median and interquartile range and were compared using the Mann–Whitney U test. Categorical variables are presented as numbers and percentages and were compared using the chi-square test or Fisher's exact test, as appropriate.

Because this was a non-randomized historical cohort study with a limited sample size, the primary analysis was exploratory and descriptive. Individual adverse-event categories were considered exploratory components of the composite primary outcome rather than independent confirmatory endpoints. No causal inference was made. A two-sided *P* value < 0.05 was considered statistically significant, and findings were interpreted as associations between pathway implementation and clinical outcomes rather than evidence of causality.

## 3 Results

### 3.1 Baseline characteristics

A total of 63 children who underwent elective ERCP were included in the study. Of these, 30 children were managed under routine perioperative management and 33 were managed under the risk-stratified perioperative safety pathway. There were no statistically significant differences between the two groups in sex distribution, age, body weight, or disease type (all *P* > 0.05), indicating that the baseline demographic and disease characteristics were broadly comparable between groups (Table 1).

**Table 1:** Baseline demographic and disease characteristics of children undergoing elective ERCP.

Indicator	Routine management group (*n* = 30)	Risk-stratified pathway group (*n* = 33)	*P* value
Male/female, n	16/14	19/14	0.855
Age, years	8.9 ± 2.4	9.7 ± 2.6	0.083
Body weight, kg	32.5 ± 6.8	34.8 ± 6.5	0.061
Disease type			0.903
Choledocholithiasis, *n* (%)	20 (66.7)	19 (57.6)	
Biliary stenosis, *n* (%)	6 (20.0)	7 (21.2)	
Others, *n* (%)	4 (13.3)	7 (21.2)	

Data are presented as mean ± SD or *n* (%). ERCP, endoscopic retrograde cholangiopancreatography; SD, standard deviation.

### 3.2 Postoperative recovery outcomes

Children in the risk-stratified pathway group showed improved postoperative recovery indicators compared with those in the routine management group. The time to first flatus was significantly shorter in the pathway group than in the routine management group (20.3 ± 5.1 vs. 27.5 ± 6.2 h, *P* < 0.01). The time to resumption of oral intake was also earlier in the pathway group (33.6 ± 7.9 vs. 44.2 ± 8.7 h, *P* < 0.01). In addition, the length of hospital stay was shorter in the pathway group (5.2 ± 1.8 vs. 7.5 ± 2.1 days, *P* < 0.01) (Table 2).

**Table 2:** Comparison of postoperative recovery outcomes.

Indicator	Routine management group (*n* = 30)	Risk-stratified pathway group (*n* = 33)	*P* value
Time to first flatus, h	27.5 ± 6.2	20.3 ± 5.1	<0.01
Time to resumption of oral intake, h	44.2 ± 8.7	33.6 ± 7.9	<0.01
Length of hospital stay, days	7.5 ± 2.1	5.2 ± 1.8	<0.01

Data are presented as mean ± SD.

### 3.3 In-hospital perioperative adverse events

The proportion of patients with at least one in-hospital perioperative adverse event was lower in the risk-stratified pathway group than in the routine management group (24.2% vs. 53.3%, *P* = 0.017). Among individual adverse-event components, nasobiliary drainage-related complications were less frequent in the pathway group (6.1% vs. 23.3%, *P* = 0.046). These complications included tube obstruction, displacement, or twisting. No statistically significant differences were observed between groups in post-ERCP pancreatitis, hyperamylasemia, biliary infection, bleeding, perforation, respiratory complications, or anesthesia-related adverse reactions (all *P* > 0.05) (Table 3).

**Table 3:** Comparison of in-hospital perioperative adverse events.

Adverse event	Routine management group (*n* = 30)	Risk-stratified pathway group (*n* = 33)	*P* value
Post-ERCP pancreatitis	2 (6.7)	1 (3.0)	0.498
Hyperamylasemia	2 (6.7)	1 (3.0)	0.498
Biliary infection	1 (3.3)	1 (3.0)	0.945
Bleeding	1 (3.3)	1 (3.0)	0.945
Perforation	1 (3.3)	0 (0.0)	0.290
Nasobiliary drainage-related complications	7 (23.3)	2 (6.1)	0.046
Respiratory complications	1 (3.3)	1 (3.0)	0.945
Anesthesia-related adverse reactions	1 (3.3)	1 (3.0)	0.945
Patients with ≥1 perioperative adverse event	16 (53.3)	8 (24.2)	0.017

Data are presented as *n* (%). Chi-square test or Fisher's exact test was used as appropriate. The final row represents the patient-level composite primary outcome; individual categories are exploratory components and may overlap. ERCP, endoscopic retrograde cholangiopancreatography.

### 3.4 Caregiver-reported care experience

Caregiver-reported care experience scores were higher in the risk-stratified pathway group than in the routine management group across all assessed domains, including professional care, communication and health education, psychological support, care attitude, and overall care experience (all *P* < 0.01). These findings suggest that implementation of the structured pathway was associated not only with improved clinical recovery, but also with better caregiver-perceived perioperative care quality (Table 4).

**Table 4:** Comparison of caregiver-reported care experience scores.

Domain	Routine management group (*n* = 30)	Risk-stratified pathway group (*n* = 33)	t value	*P* value
Professional care	3.8 ± 0.6	4.5 ± 0.4	7.51	<0.01
Communication and health education	3.7 ± 0.7	4.6 ± 0.5	8.10	<0.01
Psychological support and reassurance	3.6 ± 0.8	4.5 ± 0.5	6.85	<0.01
Care attitude and responsiveness	3.9 ± 0.6	4.6 ± 0.4	6.55	<0.01
Overall care experience	3.8 ± 0.7	4.7 ± 0.3	8.42	<0.01
Total average score	3.8 ± 0.6	4.6 ± 0.4	8.59	<0.01

Data are presented as mean ± SD.

## 4 Discussion

In this single-center real-world cohort study, implementation of a risk-stratified perioperative safety pathway was associated with improved short-term outcomes in children undergoing elective ERCP. Children managed under the pathway showed faster recovery of gastrointestinal function, earlier resumption of oral intake, shorter hospital stay, fewer patient-level in-hospital adverse events, and better caregiver-reported care experience. The most clinically interpretable reduction was observed in nasobiliary drainage-related complications, including tube obstruction, displacement, and twisting. These findings suggest that a structured perioperative safety pathway may provide added value in pediatric ERCP by improving risk communication, early warning recognition, and device-related postoperative management.

The pathway evaluated in this study was not intended to replace technical prevention of ERCP-related complications. Complications such as post-ERCP pancreatitis, bleeding, and perforation are strongly influenced by patient-related and procedure-related factors, including cannulation difficulty, pancreatic duct manipulation, sphincterotomy, and anatomical variation (5, 6). A ward-based safety pathway may not completely prevent these events from occurring, but it may improve early recognition, timely escalation, and standardized responses once warning signs appear. This may partly explain why the present study observed a reduction in the composite patient-level adverse-event outcome but did not show statistically significant differences in most individual biological or procedure-related complications.

The reduction in nasobiliary drainage-related complications is the most procedure-specific and operationally plausible finding. Nasobiliary drainage tubes require continuous postoperative attention, including assessment of tube fixation, patency, drainage characteristics, twisting, displacement, compression, and accidental traction. In children, these problems may be more frequent because of poor tolerance, movement during recovery, limited cooperation, and caregiver unfamiliarity with tube protection. Compared with biological complications such as pancreatitis, drainage-related events are more directly influenced by structured monitoring, standardized device care, and caregiver education. The observed reduction in tube-related complications therefore provides a reasonable signal that the pathway may have improved a nursing-sensitive but medically relevant safety outcome.

The results also align with the broader concept that perioperative safety improvement depends on system-level standardization rather than isolated clinical judgment. The World Health Organization Surgical Safety Checklist and subsequent comprehensive surgical safety systems showed that structured communication, checklist-based processes, and standardized perioperative workflows can reduce complications and improve patient outcomes (16, 17). Although ERCP is an endoscopic rather than open surgical procedure, the underlying safety principles are similar: key risk information should be identified before the procedure, communicated across teams, and translated into postoperative monitoring and response plans. In this study, the pathway functioned as a procedure-specific safety system connecting preoperative assessment, intra-procedural risk handover, post-procedural observation, and discharge-oriented caregiver education.

Another important aspect of this study is that the pathway was implemented in routine clinical practice rather than in a tightly controlled prospective trial. This real-world design increases clinical relevance but also introduces methodological challenges. Quality improvement interventions are complex, context-dependent, and influenced by local workflow, team training, leadership, and implementation fidelity. The SQUIRE 2.0 guidelines emphasize that quality improvement studies should clearly describe the intervention, context, mechanisms, measures, and limitations when assessing healthcare safety interventions (18). In the present study, the intervention was multidimensional, and its effect may have resulted from the combined influence of risk stratification, structured handover, early-warning monitoring, device management, and caregiver participation rather than from any single component.

The improvements in time to first flatus, time to oral intake, and length of hospital stay may reflect more efficient postoperative recovery under a structured care process. These outcomes are consistent with the general principles of enhanced recovery after surgery, in which standardized perioperative pathways, early functional recovery, patient and family education, and coordinated multidisciplinary care are used to improve postoperative outcomes. Although ERCP is not equivalent to abdominal surgery, pediatric enhanced recovery studies have shown that structured perioperative pathways can reduce length of stay, shorten time to feeding, and improve recovery-related outcomes in children undergoing abdominal operations (19). The present findings suggest that similar pathway-based concepts may be adapted to pediatric endoscopic procedures, particularly those requiring anesthesia, postoperative fasting, drainage management, and complication surveillance (20).

Caregiver-reported care experience was also improved in the pathway group. This finding should be interpreted cautiously because caregiver experience is subjective and may be influenced by communication style, expectations, and non-blinded care delivery. Nevertheless, in pediatric perioperative practice, caregiver participation is not merely a satisfaction issue; it is also part of the safety system. Caregivers help observe pain, vomiting, fever, behavioral changes, and tube-related problems, particularly in younger children who cannot accurately describe symptoms. Structured education and repeated communication may therefore improve not only perceived care quality but also the reliability of postoperative observation.

Several limitations must be acknowledged. First, this was a single-center retrospective study with a relatively small sample size, and the findings should be considered exploratory. Second, the two groups were defined according to historical implementation periods. Therefore, temporal bias related to increasing team experience, improved procedural skills, changes in case complexity, or subtle changes in perioperative practice cannot be excluded. Third, detailed procedural variables such as cannulation difficulty, pancreatic duct injection, sphincterotomy, stent placement, procedure duration, and anesthesia duration were not fully analyzed in the current dataset. These variables may confound the association between pathway implementation and outcomes. Fourth, the composite adverse-event outcome combined heterogeneous events, some of which may be more modifiable by perioperative management than others. Fifth, caregiver-reported experience was assessed before discharge and may have been influenced by expectation and non-blinded implementation. Finally, the study only evaluated in-hospital outcomes, and delayed adverse events after discharge were not systematically captured.

Despite these limitations, the study provides preliminary real-world evidence that a structured, risk-stratified perioperative safety pathway may be feasible and clinically meaningful in pediatric ERCP. Future studies should use prospective multicenter designs, collect detailed procedural risk variables, apply adjusted analyses or propensity-score methods when sample size permits, and evaluate both in-hospital and post-discharge outcomes. Development of a standardized pediatric ERCP perioperative safety checklist may further improve reproducibility and facilitate comparison across centers.

## 5 Conclusions

In this single-center retrospective historical cohort study, children treated during the risk-stratified pathway period had shorter postoperative recovery times, a lower observed proportion of in-hospital perioperative adverse events, and higher caregiver-reported care experience scores than children treated during the earlier routine-management period. Because pathway implementation coincided with calendar time, these findings may also have been influenced by operator experience, drainage-device selection, and other unmeasured temporal changes. The results should therefore be interpreted as exploratory associations rather than evidence of causality. Prospective multicenter studies are needed to validate the effectiveness and generalizability of this pathway in pediatric ERCP.

## Data Availability

The raw data supporting the conclusions of this article will be made available by the authors, without undue reservation.
